# Foot health-related quality of life in hemophiliacs: A case-control study

**DOI:** 10.7150/ijms.48705

**Published:** 2020-08-29

**Authors:** Ana María Jiménez-Cebrián, Daniel López-López, Ricardo Becerro-de-Bengoa-Vallejo, Marta Elena Losa-Iglesias, Emmanuel Navarro-Flores, Marta San-Antolín, César Calvo-Lobo, Patricia Palomo-López

**Affiliations:** 1 Department Nursing and Podiatry, Faculty of Health Sciences, University of Málaga, c/ Arquitecto Francisco Peñalosa 3, Ampliación del Campus de Teatinos, 29071 Málaga, Spain, Instituto de Investigación Biomédica de Málaga (IBIMA) https://orcid.org/000-0002-8634-7114.; 2 Research, Health and Podiatry Group. Department of Health Sciences, Faculty of Nursing and Podiatry. Universidade da Coruña, Ferrol, Spain.; 3 Facultad de Enfermería, Fisioterapia y Podología. Universidad Complutense de Madrid, Spain.; 4 Faculty of Health Sciences. Universidad Rey Juan Carlos, Spain.; 5 Faculty of Nursing and Podiatry, Department of Nursing. University of Valencia. Frailty Research Organizaded Group (FROG).; 6 Department of Psychology, Universidad Europea de Madrid, Villaviciosa de Odón, Madrid, Spain.; 7 University Center of Plasencia. Universidad de Extremadura, Spain.

**Keywords:** measurement/psychometrics, quality of life, chronic pain

## Abstract

**Background:** Haemophilia is considered as a chronic genetic disease related with alteration in coagulation mechanism which affects to health related quality of life (HQoL).

**Purpose:** The goal compared marks of HQoL, in haemophiliacs with respect non haemophilic subjects.

**Methods:** A population of 74 subjects, were recruited from association of haemophilic illness separated in haemophilic subjects (n = 37) and no haemophilic (n = 37). For subjects who suffered haemophilia were enlisted from the association of haemophilic illness after a seminar of 45 minutes to them and to their relatives about foot health. Control subjects, were recruited from their relatives who live with the patient. The marks of the Foot Health Status Questionnaire Spanish S_FHSQ sub-scales were recompiled.

Results: All S_FHSQ domains as foot pain, foot function, tootwear, general foot health, general health, physical activity and social capacity showed lower scores in the haemophilic than non-haemophilic group (*P* <0.01) except for vigour (*P* = 0.173). Regarding the rest sub-scale marks of S_FHSQ, showed no significant difference *P* <0.01.

**Conclusion:** Subjects with a haemophilia showed significant worse foot QoL in all S_FHSQ domains except vigour domain compared with non-haemophilic subjects.

## Introduction

Haemophila is an incurable genetic disease, the main symptom is the deficit or non-existance of coagulant factors VIII or IX (Haemophilia type A and type B, respectively). This chromosomic disorder, affects only men, the global prevalence is estimated in 5000 males in case of type A and in 25,000 for type B 1. The incidence represents 1 case per 10.000 men, while hemophilia B incidence are 1 case every 30,000 men 2,3.

In case of severe Haemophilia the main characteristic is a spontaneous bleeding, even ostheo articular structures and internal organs, which can be affects to health Qol.

Nowadays treatment consists in routine administration of deficient factor, 2 to 3 times a week, preventing spontaneous bleeding 4.

Regarding health related Qol, it can be defined as “an individual's or group's perceived physical and mental health over time” 5.

Continuous joint haemorrhagic cause ostheo articular disorders due to cartilage destruction, in consequence, appears an haemophilic arthropathy which can increase a poor HQol with associated symptom likely foot disorders, and generate great disability 6.

In fact as a consequence of this alteration, can appear an arthropatic process. Characteristic of the affecting structures involves 7,8.

Also foot disorders can affect to HQoL, in fact, prior researches showed that hallux deformation subjects have poorer levels (low score) in HQoL as a consequence of this problem 9.

Furthermore, this disability affected some psychological aspects including depression symptoms 10.

For this reason haemophilic subjects can be also affected in their general health especially in aspects related to HQoL on their foot disorders, as the case of plantar fasciitis 11 or psychosocial aspects 12,13. The main purpose of this study was to compare both foot and general health-related quality of life between haemophiliac and healthy subjects. We hypothesized that haemophiliac may present an impaired quality of life related to foot and general health.

## Methods

### Sample and Design

An observational case-control study was performed wherein subjects were recruited from the association of haemophilic illness from Málaga, Cantabria and Aragón (Spain).

Study subjects consist of haemophilic subjects (n = 37) and non-haemophilic (n = 37) Patients were recruited by a consecutive sampling method using a successive and non-aleatorized sample method. Subjects who suffered haemophilia were enlisted from the association of haemophilic illness after a seminar of 45 minutes to them and to their relatives about foot health. Control subjects, were recruited from their relatives who live with the patient.

Inclusion criteria: 1) over 18 years, 2) haemophilic subjects' case group, and subjects without haemophlia or health disorders (control group), and 3) subjects who authorized their consent in signing the form.

Subjects exclusion criteria were: 1) another different genetic disease than haemophilia, 2) history of foot surgery or trauma in lower limb, 3) antecedents of haematologic disease, 4) no acquiescence in written form and finally difficulties understanding the instructions for completing the research course. Cases and control were matched by age.

### Plan of action

Outcomes contained ordinary topics relative to generic health status, about demography aspects: years, height and weight. Subjects also completed S_FHSQ 14.

After that, informants filled out the Foot Health Status Questionnaire (FHSQ) 15. This self-administered questionnaire on health-related quality of life is intended specifically for the foot which is recognised as a validated test 16,17. Foot-specific and general health-related quality of life was assessed by using the Foot Health Status Questionnaire (version 1.03) 17, which comprised of three main sections. Section 1 consists of 13 questions reflecting four foot health-related domains (**Table 1**): foot pain, foot function, footwear, and general foot health. This section has demonstrated a high degree of content, criterion, and construct validity (Cronbach α= 0.89-0.95) and high retest reliability (intraclass correlation coefficient = 0.74-0.92), 17 and it has been shown to be the most appropriate measure of health-related quality of life for chronic plantar heel pain population 18.

Each domain has a specific number of questions (**Table 2**). In particular, 4 regarding pain, 4 on function, 3 on footwear and 2 on general foot health. The assessment of pain and function is based on physical phenomenon in which the evaluation of footwear uses practical aspects related to availability and the comfort of the shoes, while the perception of the foot's general health is based on the patients' self-assessment of the state of their feet. Each question allows several answers and these are placed on a Likert-type ordinal scale (words or phrases corresponding to a numeric scale). The descriptors for these scales vary for each domain and the person completing the questionnaire has to choose only one response, whichever is thought to be the most appropriate. The questionnaire does not provide a global score, but rather generates an index for each domain. In order to obtain these indices, the responses are analysed by a computer program (The FHSQ, Version 1.03) which, after processing the data, gives a score ranging from 0 to 100. A 0 score represents the worst state of health for the foot and 100 is the best possible condition. Furthermore, the software also provides graphical images of the outcomes.

### Ethics procedure

Ethics Committee of the Universidad de Extremadura number 263/2019 Spain were obtained, also subjects given their permission to participate signing informed consent, before to include them at the study. The study was developed follow the ethical principles for Clinical Research 19.

### Sample size calculation

The estimates of sample size was developed using two different groups with individual samples using G*Power software and were built accordingly foot function sub-scale of S_FHSQ results from an exploratory research (n = 20) tailed, 10 haemophilic subjects then 10 non haemophilic linked in pairs subjects. Also, the effect size 0.40, using a two-tailed hypothesis, 0.01 alpha error potential probability [probability of 1- betha error] of 0.99 and assignment proportion [N2/N1] of one were employed for the computation of the sample dimension. Regarding, the composition sample dimension was 74 subjects, 37 haemophiliacs and 37 non haemophilic linked in pairs subjects, was determined with an actual power of 0.909.

### Statistics

For the normality distribution of the variables, the Kolmogorov-Smirnov test was used, and data were considered as normally distributed if *P* >0.05. Regarding the results of quantitative variables, data was described as mean and standard deviation (SD) with IC95% and range showing maximum and minimum values in terms of their median, interquartile range (IR), and IC95% Minim and maxim (range) values. Parametric data were determined using mean, standard deviation (SD), and minimal and maxim (range) values.

A comparison of the quantitative data between groups to find out significant differences were checked using an Independent student *t*-test for parametric data and Mann-Whitney U test for non-parametric data.

All analyses were considered statistically significant when the *p*-value < 0.01 with a 99% confidence interval (CI). Statistical analyses were developed with SPSS (V.26.0, Chicago, IL, United States).

## Results

All data showed a normal distribution (*P* >0.05) except for foot pain, foot function, general foot health, general health, physical activity and social capacity (*P* >0.05).

A total population of 77 subjects were included in the study and 3 haemophilic participants and their matched control were excluded because did not sign the informed consent. So, finally 74 participants were included in the study, 37 subjects in the haemophilic group and 37 subjects in the non-haemophilic group.

Descriptive data of participants are showed in **Table 1** and there were no significant differences between groups (*P* >0.01).

### Outcome Measurements

All S_FHSQ domains showed lower scores in the haemophilic than non-haemophilic group (*P* <0.01), except for the vigour domain (*P* = 0.173).

## Discussion

The investigation objective compared scores general and foot health, frequently in relation with QoL in haemophiliac subjects with respecting non-haemophiliac subjects.

Consequently, foot management is considered as main objective in haemophilic subjects as a consequence of the expanding of ostheo articular disorders in different body region with a great health impact in haemophilic subjects, regarding the previous research which may be considered as a public health problem according the results of Peyvandi 2 who analyse several kind of haemophilia treatment or in the other hand the review of Bertamino 20 who evaluated treatment in children. Furthermore, a research had studied the perception of quality of life concluded that damage in haemophilic Qol and also releated to foot disorders on fibromyalgia women patients 21-23.

However, there is a deficient of research of this disorder and the influence of the QoL coupled to foot well-being and state of general health. As a consequence, the finding of our study corroborate that haemophilic presented damage on the QoL coupled to foot well-being and general foot health, compared to the non-haemophiliac group.

Regarding these finding, the score health damage suggest relevancy. That situation emphasises a call for foot checking it must develop by different clinical professionals, beside the foot medical management, to avoid disorders presence and feet estate. Accordingly similar research in subjects with foot disorders 24,25, foot pain 26, and neurological disorders such as Alzheimer disease 27, our results also founded that shoes and physical activities, could take part an essential aspect in the QoL respect in well-being foot in haemophilic subjects. That situation can be the main important question that would allow the development of the well-being QoL and in haemophilic population. With respect to our discoveries in foot QoL damage in this kind of subjects, subsequence involvement researches must check the repercussions of foot function on the QoL with respect to well-being foot in haemophilic population.

Regarding depressive symptoms it was found that comparing repercussions of the findings we obtained to different studies was impracticable, due to the different methods applied in previous studies, besides, we have not been competent to discover any QoL work in haemophilic foot health previously published.

Finally, the present research had some limitations. Heterogeneous characteristics of the sample, subjects who lives in several environment and bigger sample dimension must be taking into account to intensify the soundness of the work and would be helpful to identify foot disabilities in distinct haemophilics, as a diagnostic tool related to damage in well- being foot and QoL.

Moreover, the consecutive sampling method may be the main limitation of this research.

## Conclusion

These results show that subjects with haemophilia presented a lower foot QoL, in the following domains: foot pain, foot function, footwear, general foot health, general health, physical activity and social capacity except vigour compared with healthy subjects. Furthermore, general health status, footwear and physical activity, which appearance to be linked to haemophilia condition. In consequence, adequate foot and checking care evaluation of the overall well- being foot could be essential to keep way the spread of disabilities, ostheo aritcular disorders or haemorragic symptom across the disease evolution of the haemophilic.

## Figures and Tables

**Table 1:** Basic domains of foot health assessed by the Foot Health Status Questionnaire. Section 1 domains

Domain	Item	Theoretical Construct	Meaning of Lowest Score (0)	Meaning of Highest Score (100)
Foot pain	4	Type, severity and duration. Evaluation of foot pain in terms of type of pain, severity and duration	Extreme pain in the feet and significant if acute in nature	Free from pain, no discomfort
Foot function	4	Evaluation of the feet in terms of impact on physical functions	Severely limited for the performance of numerous physical activities due to their feet, such as walking, working and moving about	Patients are able to carry out all physical activities desired, such as walking, working and climbing stairs
General foot health	2	Self-perception of the feet (assessment of body image with respect to feet)	Perception of poor condition and status of the feet	Perception of excellent condition and status of the feet
Footwear	3	Lifestyle relating to footwear and feet	Great limitations to find suitable footwear	No problem obtaining suitable footwear. No limitations with respect to footwear

**Table 2:** 13 questions of the Foot Health Status Questionnaire that assess 4 health domains of the feet: pain, function, general health and footwear

No.	Question
1.	What level of foot pain have you had during the past week?
2.	How often have you had foot pain?
3.	How often did your feet ache?
4.	How often did you get sharp pains in your feet?
5.	Have your feet caused you to have difficulties in your work or activities?
6.	Were you limited in the kind of work you could do because of your feet?
7.	How much does your foot health limit you walking?
8.	How much does your foot health limit you climbing stairs?
9.	How would you rate your overall foot health?
10.	It is hard to find shoes that do not hurt my feet.
11.	I have difficulty in finding shoes that fit my feet.
12.	I am limited in the number of shoes I can wear.
13.	In general, what condition would you say your feet are in?

Section 2 includes questions that reflect four general health-related domains general health, physical activity, social capacity, and vigor (**Table 3**). The domains and questions in this section are largely adapted from the Medical Outcomes Study 36-Item Short-Form Health Survey, which has been validated for use in the Australian population. Specific questions of the Foot Health Status Questionnaire that assess section II domains are shown in **Table 4.**

**Table 3:** Definitions of the Foot Health Questionnaire. Section 2 Domains

Domain	Theoretical Construct	Meaning of Lowest Score (0)	Meaning of Highest Score (100)
General health	Evaluation of subject´s self-reportedhealth status	Poor perception of helth status	Very good general health status
Physical activity	Evaluation of ability in terms of impact on physical function	Severely limited in performing a broad raange of physical activities	Can perform all desired physical activitues with no impairment or disability
Social Capacity	Self-perceptions of ability to socially interact	Limited ability to interact without problems, socially isolated	Good ability to interact socially and experience no isolation
Vigor	Lifestyle issues to perceived energyand activity participation	Lacks energy to do things	No problems with energy levels

**Table 4:** Questions of the Foot Health Status Questionnaire that assess section 2 domains

No.	Question
14.	In general, how would you rate your health?
15.	The following questions ask about activities you might do during a typical day. Does your health now limit you in these activities? **a.** Vigorous activities, such as running, lifting heavy objects, or (if you wanted to) your ability to participate in strenuous sports. **b.** Moderate activities, such as cleaning the house, lifting a chair, playing golf or swimming. **c.** Lifting or carrying bags of shopping. **d.** Climbing a steep hill. **e.** Climbing one flight of stairs. **f.** Getting up from a sitting position. **g.** Walking more than a kilometer. **h.** Walking one hundred meters. **i.** Showering or dressing yourself.
16.	To what extent has your physical health or emotional problems interfered with your normal social activities with family, friends, neighbors or social groups?
17.	These questions are about how you feel during the past month. For each question, please give the one answer that comes closest. How much of the time during the past 4 weeks: **a.** Did your feet get tired? **b.** Did you have a lot of energy? **c.** Did your feet feel worn out? **d.** Did you feel full of life?
18.	During the past 4 weeks, how much of the time has your emotional problems or physical health interfered with your social activities (like visiting with friends, relatives, etc.)?
19.	How true or false is each of the following statements for you? **a.** I seem to get sick a little easier than other people. **b.** I am as healthy as anybody I know. **c.** I expect my health to get worse. **d.** My health is excellent.

Section 3 collects socioeconomic status, comorbidity, service utilization and satisfaction information and their medical record.

**Table 5:** Descriptive data of haemophilic and non-haemophilic subjects

Descriptive Data	Total Group (*N* = 74), mean ± SD (Range)	Haemophilic (*n* = 37), mean ± SD (Range)	Non haemophilic (*n*=37), mean ± SD (Range)	*P* value
Birth (years)	43.51 ± 16.66 (18-80)	43.51 ± 16.77 (18-80)	42.50 ± 30.75 (18-80)	1.000*
Weight (kg)	78.00 ± 19.00 (56-140)	77.00 ± 19.50 (56-140)	78.00 ± 18.50 (61-100)	0.858*
Height (m)	1.74 ± 0.07 (1.56-1.90)	1.74 ± 0.08 (1.56-1.85)	1.74 ± 0.07 (1.57-1.90)	0.840*
BMI (kg/m^2^)	26.26 ± 3.83 (19.30-40.90)	26.45 ± 4.38 (19.30-40.90)	26.08 ± 3.24 (20.90-33.20)	0.677*

Abreviations: BMI, body mass index. standard deviation, *, Independent Student t -test were employed. P value <0.01with a 99% confidence interval was considered statistical significant.

**Table 6:** S_FHSQ domains scores between haemophilic and non haemophilic subjects

Outcome Measurements, S FHSQ	Total Group (*N* = 74), Mean±SD (Range)	Haemophilic (*n* = 37), Mean±SD (Range)	Non haemophilic (*n* = 37), Mean±SD (Range)	*P* value (Haemophilic vs. non-haemophilic)
Foot pain	74.12 ± 34.38 (0-100)	60.00 ± 36.56 (0-100)	90.62 ± 21.88 (54.38-100)	<0.001^†^
Foot function	75.00 ± 45.31 (6.25-100)	56.25 ± 40.63 (6.25-100)	100.00 ± 15.24 (25-100)	<0.001^†^
Footwear	51.80 ± 34.90 (0-100)	38.51 ± 32.17 (0-100)	65.09 ± 32.73 (0-100)	0.001*
General foot health	60.00 ± 60.00 (0-100)	25.00 ± 60.00 (0-92.50)	85.00 ± 40.00 (25-100)	<0.001^†^
General health	70.00 ± 42.50 (10-100)	50.00 ± 40.00 (10-100)	80.00 ± 30.00 (50-100)	<0.001^†^
Physical activity	77.77 ± 44.44 (16.67-100)	55.55 ± 27.78 (16.67-100)	100.00 ± 16.67 (38-89-100)	<0.001^†^
Social capacity	87.50 ± 25.00 (12.50-100)	75.00 ± 31.25 (12.50-100)	100.00 ± 12.50 (62.50-100)	<0.001^†^
Vigour	68.58 ± 31.25 (31.25-100)	65.54 ± 18.72 (31.25-100)	71.62 ± 19.29 (37.50-100)	0.173*

Abbreviations: S_FHSQ, Foot Health Status Questionnaire. *, Independent Student t -test were employed. †, Mann Whitney U test was employed. P value <0.01with a 99% confidence interval was considered statistical significant.

## References

[1] Forneris E, Andreacchio A, Pollio B, Mannucci C, Franchini M, Mengoli C (2016). Gait analysis in children with haemophilia: First Italian experience at the Turin Haemophilia Centre. Haemophilia.

[2] Peyvandi F, Garagiola I, Young G (2016). The past and future of haemophilia: diagnosis, treatments, and its complications. Vol. 388, The Lancet. Lancet Publishing Group.

[3] Fiorillo L, De Stefano R, Cervino G, Crimi S, Bianchi A, Campagna P (2019). Oral and psychological alterations in haemophiliac patients. Vol. 7, Biomedicines. MDPI AG.

[4] McLaughlin P, Chowdary P, Woledge R, McCarthy A, Mayagoitia R (2013). The effect of neutral-cushioned running shoes on the intra-articular force in the haemophilic ankle. Clin Biomech.

[5] Moriarty DG, Zack MM, Kobau R (2003). The Centers for Disease Control and Prevention's Healthy Days Measures - population tracking of perceived physical and mental health over time. Health Qual Life Outcomes.

[6] Fijnvandraat K, Cnossen MH, Leebeek FWG, Peters M (2012). Diagnosis and management of haemophilia. Vol. 344, BMJ (Online).

[7] Stephensen D, Tait R, Brodie N, Collins P, Cheal R, Keeling D (2009). Changing patterns of bleeding in patients with severe haemophilia A. Haemophilia.

[8] Lobet S, McCarthy A, Hermans C, Peerlinck K, Matricali GA, Staes F, et al. Biomechanical markers and theoretical concepts related to haemophilic ankle and subtalar joint arthropathy: introducing the term 'haemophilic tarsal pan-arthropathy.' Vol. 23, Haemophilia. Blackwell Publishing Ltd; 2017. p. e250-8 10.1111/hae.13202 28306191

[9] López DL, Fernández JMV, Iglesias MEL, Castro CÁ, Lobo CC, Galván JR (2016). Influence of depression in a sample of people with hallux valgus. Int J Ment Health Nurs.

[10] Beck AT, Ward CH, Mendelson M, Mock J, Erbaugh J (1961). An Inventory for Measuring Depression. Arch Gen Psychiatry.

[11] Iannone M, Pennick L, Tom A, Cui H, Gilbert M, Weihs K (2012). Prevalence of depression in adults with haemophilia. Haemophilia.

[12] Witkop ML, Lambing A, Nichols CD, Munn JE, Anderson TL, Tortella BJ (2019). Interrelationship between depression, anxiety, pain, and treatment adherence in hemophilia: Results from a US cross-sectional survey. Patient Prefer Adherence.

[13] Fiorillo L, Stefano R De, Cervino G, Crimi S, Bianchi A, Campagna P, et al. Haemophiliac Patients.:1-13

[14] Cuesta-Vargas A, Bennett P, Jimenez-Cebrian AM, Labajos-Manzanares MT (2013). The psychometric properties of the Spanish version of the Foot Health Status Questionnaire. Qual Life Res.

[15] Palomo-López P, López-López D, Becerro-De-Bengoa-Vallejo R, Losa-Iglesias ME, Rodríguez-Sanz D, Fernández-Carnero J (2019). Concurrent validity of the foot health status questionnaire and study short form 36 for measuring the health-related quality of life in patients with foot problems. Med.

[16] Bennett PJ, Patterson C, Wearing S, Baglioni T (1998). Development and validation of a questionnaire designed to measure foot-health status. J Am Podiatr Med Assoc.

[17] Bennett PJ, Patterson C, Dunne MP (2001). Health-related quality of life following podiatric surgery. J Am Podiatr Med Assoc.

[18] Landorf KB, Keenan A-M (2002). An evaluation of two foot-specific, health-related quality-of-life measuring instruments. Foot ankle Int.

[19] Holt GR (2014). Declaration of Helsinki—The World's Document of Conscience and Responsibility. South Med J.

[20] Bertamino M, Riccardi F, Banov L, Svahn J, Molinari A (2017). Hemophilia Care in the Pediatric Age. J Clin Med.

[21] Witkop M, Neff A, Buckner TW, Wang M, Batt K, Kessler CM (2017). Self-reported prevalence, description and management of pain in adults with haemophilia: methods, demographics and results from the Pain, Functional Impairment, and Quality of life (P-FiQ) study. Haemophilia.

[22] Buckner TW, Wang M, Cooper DL, Iyer NN, Kempton CL (2017). Known-Group validity of patient-reported outcome instruments and hemophilia joint health score v2.1 in US adults with hemophilia: Results from the pain, functional impairment, and quality of life (P-FiQ) study. Patient Prefer Adherence.

[23] Palomo-López P, Calvo-Lobo C, Becerro-de-Bengoa-Vallejo R, Losa-Iglesias ME, Rodriguez-Sanz D, Sánchez-Gómez R (2019). Quality of life related to foot health status in women with fibromyalgia: a case-control study. Arch Med Sci.

[24] López-López D, Becerro-de-Bengoa-Vallejo R, Losa-Iglesias ME, Palomo-López P, Rodríguez-Sanz D, Brandariz-Pereira JM (2018). Evaluation of foot health related quality of life in individuals with foot problems by gender: a cross-sectional comparative analysis study. BMJ Open.

[25] Rodríguez-Sanz D, Tovaruela-Carrión N, López-López D, Palomo-López P, Romero-Morales C, Navarro-Flores E Foot disorders in the elderly: A mini-review. Disease-a-Month. 2017.

[26] Navarro-Flores E, Losa-Iglesias M, Becerro-de-Bengoa-Vallejo R, López-López D, Rodríguez-Sanz D, Palomo-López P (2018). Translation and Test-Retest of the Spanish Podiatry Health Questionnaire (PHQ-S). Int J Environ Res Public Health.

[27] Navarro-Flores E, Pérez-Ros P, FM M-A, Julían-Rochina I, Cauli O (2019). Neuro-psychiatric alterations in patients with diabetic foot syndrome. CNS Neurol Disord - Drug Targets.

